# Revealing the maternal demographic history of *Panthera leo* using ancient DNA and a spatially explicit genealogical analysis

**DOI:** 10.1186/1471-2148-14-70

**Published:** 2014-04-02

**Authors:** Ross Barnett, Nobuyuki Yamaguchi, Beth Shapiro, Simon YW Ho, Ian Barnes, Richard Sabin, Lars Werdelin, Jacques Cuisin, Greger Larson

**Affiliations:** 1 Durham Evolution and Ancient DNA, Department of Archaeology, Durham University, Durham DH1 3LE, UK; 2 Department of Biological and Environmental Sciences, Qatar University, Doha, Qatar; 3 Department of Ecology and Evolutionary Biology, University of California Santa Cruz, Santa Cruz, CA 95064, USA; 4 School of Biological Sciences, University of Sydney, Sydney, NSW 2006, Australia; 5 School of Biological Sciences, Royal Holloway University of London, Egham, Surrey TW20 0EX, UK; 6 Department of Life Science, Natural History Museum, Cromwell Road, London SW7 5BD, UK; 7 Department of Palaeobiology, Swedish Museum of Natural History, Box 50007, Stockholm SE-10405, Sweden; 8 Muséum National d’Histoire Naturelle, 54 Rue Cuvier, Paris 75005, France; 9 Current Address: Centre for GeoGenetics, København Universitet, The Natural History Museum Øster Voldgade, Copenhagen 5-7 DK-1350, Denmark

**Keywords:** Barbary lion, *Panthera leo*, Extinction, Mitochondrial DNA, Ancient DNA, Phylogeography

## Abstract

**Background:**

Understanding the demographic history of a population is critical to conservation and to our broader understanding of evolutionary processes. For many tropical large mammals, however, this aim is confounded by the absence of fossil material and by the misleading signal obtained from genetic data of recently fragmented and isolated populations. This is particularly true for the lion which as a consequence of millennia of human persecution, has large gaps in its natural distribution and several recently extinct populations.

**Results:**

We sequenced mitochondrial DNA from museum-preserved individuals, including the extinct Barbary lion (*Panthera leo leo*) and Iranian lion (*P. l. persica*), as well as lions from West and Central Africa. We added these to a broader sample of lion sequences, resulting in a data set spanning the historical range of lions. Our Bayesian phylogeographical analyses provide evidence for highly supported, reciprocally monophyletic lion clades. Using a molecular clock, we estimated that recent lion lineages began to diverge in the Late Pleistocene. Expanding equatorial rainforest probably separated lions in South and East Africa from other populations. West African lions then expanded into Central Africa during periods of rainforest contraction. Lastly, we found evidence of two separate incursions into Asia from North Africa, first into India and later into the Middle East.

**Conclusions:**

We have identified deep, well-supported splits within the mitochondrial phylogeny of African lions, arguing for recognition of some regional populations as worthy of independent conservation. More morphological and nuclear DNA data are now needed to test these subdivisions.

## Background

Understanding the population history of a species is critical, not only to gain insight into past evolutionary processes, but also as a means of predicting responses to future environmental change [1-4]. Estimates of demographic history are increasingly reliant on genetic data, particularly in many tropical regions where the mammalian fossil record is constrained by poor preservation of bone (e.g. [5]). However, large carnivoran species have usually experienced historical persecution, and now occupy only a small subset of their former range [6-9]. This is problematic because sampling solely from extant populations provides an incomplete and potentially misleading view of the overall demographic history of a species [10,11] Under such circumstances, it is particularly important to incorporate historically collected samples into the data set, in order to achieve a more complete record of the spatial distribution of past genetic diversity [12,13].

The lion (*Panthera leo, sensu lato*) had one of the largest geographical distributions of any terrestrial mammal during the Late Pleistocene, ranging from southern Africa (*Panthera leo ssp.*), through northern Eurasia (*Panthera leo spelaea*), to Central America (*Panthera leo atrox*) [14]. However, studies of the lion’s population history are made challenging by its sparse African Pleistocene fossil record [15], as well as large-scale, human-driven extirpations in many parts of its historical range [16,17]. Widespread hunting and anthropogenic changes to lion habitat are continuing to reduce lion populations across their entire range [6,18]. On the Kathiawar Peninsula in India, the Asian lion subspecies *P. leo persica* survives as a population of only ~400 individuals and has been classified as “Endangered” by the IUCN [18]. Lions in Africa have experienced a suspected ~30% loss of the free-ranging population in the past two decades and are now classified as “Vulnerable” by the IUCN [18]. Of special concern are the populations in West and Central Africa, which may be close to extinction in the wild and are underrepresented in the captive zoo population [19-21].

Despite this regional variation in population dynamics and decline, all African lions are grouped under one conservation heading as *P. leo leo*[18]. A lack of robust data is a hindrance to producing an integrated and comprehensive evolutionary history for *P. leo* upon which to test assumptions about evolutionarily significant units [22]. Therefore, a better understanding of the evolutionary relationships among modern lion populations is critical to developing evidence-based plans for their conservation and management.

Previous analyses of short fragments of the mitochondrial control region (hypervariable region I) from modern and recently extinct lion populations revealed several distinct mitochondrial clades, but were unable to provide strong statistical support for these groupings [19,20,23,24]. Nonetheless, the extinct North African Barbary lion and the Asian lion (including the extinct Middle Eastern population) clearly form a mitochondrial group that is distinct from all sub-Saharan African lions [19,23]. This pattern is supported by morphological analyses that show similarities in cranial characters among North African and Asian lions, and separate these two populations from sub-Saharan African lions [25,26]. Relationships among modern lions have also been studied using longer sequences of mitochondrial cytochrome *b* (*cytb*) and nuclear DNA sampled from extant populations in eastern and southern Africa [27-29] and West and Central Africa [20].

The results from genetic analyses have generally supported the accepted geographical subspecies designations [6,30], though confusion has occasionally arisen when using voucher specimens from zoological collections to represent wild populations [31]. The Moroccan Royal Lion collection, for example, has been used as a proxy for extinct North African Barbary lions [e.g. 28], despite the fact that mitochondrial studies have demonstrated that these samples cluster with lions from Central Africa, rather than North Africa [19,20]. These studies have provided crucial information for understanding the phylogeographical relationships among modern lions. However, they were based on very short fragments of mitochondrial DNA, or samples drawn only from the extant populations, or data sets that included individuals with questionable geographical origins. For these reasons, there is a clear need for the phylogeography of the modern lion to be investigated using a robust molecular data set that includes representatives of extinct populations.

In order to investigate the demographic and evolutionary history of African and Asian lions (*Panthera leo, sensu stricto)*, we have used a combination of ancient and modern DNA sequences. We make use of a new approach that integrates spatial and Bayesian phylogenetic analyses, allowing ancestral geographical states to be co-estimated with the phylogeny, evolutionary rate, and coalescence times [32]. We place this history into the context of environmental changes in the late Quaternary and discuss whether the current taxonomic and management paradigm reflects biological reality.

## Methods

### DNA extraction and amplification

We collected bone and tissue samples from known-origin lion specimens kept in natural history collections (Table 1) that had previously produced 130 bp sequences of the HVR1 control region [19,23,33]. We extracted DNA using an ion-exchange column method [34]. Briefly, we reduced samples of cortical bone to powder in a mikrodismembrator (Sartorius). We then digested bone powder overnight at 50°C in 2 ml of buffer (0.425 M EDTA pH8, 1 mM Tris–HCl pH8, 0.05% w/v SDS, 0.33 mg/ml Proteinase K) under constant rotation. We concentrated the digested solution to approximately 500 μl using centrifugal filters with a molecular weight cut-off of 30 kDa (Amicon® Ultra, Millipore). We passed the concentrated solution through a silica column (QIAquick®, Qiagen) following the manufacturer’s protocol, and eluted the final extract in 100 μl of TE buffer. We measured DNA concentration (Table 1) using 2 μl of extract on the Qubit® platform (Invitrogen), and stored the extracts at -20°C.

**Table 1:** All ancient samples used in the analyses with their total DNA concentration, together with information on origin and previously published control region sequences

**Extract ID**	**Element**	**Place of origin**	**Region**	**Museum accession number (Museum)**	**CytB haplotype**	**Genbank accession**	**Control region haplotype**	**Total DNA concentration (ng/μl)**
PL1	Skull	Senegal	West	A1892 (PARIS)	C	KJ545522	M4	0.529
PL2	Skull	Senegal	West	1890-490 (PARIS)	D	KJ545523	M4	2.4
PL3	Mandible	Barbary	North	A58:5827 (STOCKHOLM)	G	KJ545524	M11	3.14
PL4	Skull	Burkina Faso	West	1926-248 (PARIS)	C	KJ545525	M3	0.869
PL5	Tissue	Tunisia	North	BARBARY C (LEIDEN)	E	KJ545526	M11	10.2
PL6	Skull	North Africa	North	A7912 (PARIS)	E	KJ545527	M11	3.89
PL7	Vertebra	Algeria	West	1862-54 (PARIS)	E	KJ545528	M11	1.22
PL8	Skull	Iran	Middle East	1962-2847 (PARIS)	F	KJ545529	M10	3.92
PL9	Skull	Iran	Middle East	1962-2854 (PARIS)	F	KJ545530	M10	3.94
PL11	Mandible	Tower of London	North	1952.10.20.15 (NHM)	E	KJ545531	M11	0.146
PL12	Mandible	Tower of London	North	1952.10.20.16 (NHM)	H	KJ545532	M11	0.091
PL13	Vertebra	Sudan	Central	1995-164 (PARIS)	A	KJ545533	M8	0.59
PL15	Skull	Central African Republic	Central	1996-2516 (PARIS)	A	KJ545534	M6	5.01
PL16	Skull	Central African Republic	Central	1996-2517 (PARIS)	B	KJ545535	M6	0.304

We selected primers to amplify short (<200 bp) overlapping fragments of mitochondrial *cytb* in order to maximize the success rate of amplification from historical specimens. We designed novel primers specific to *Panthera leo* that minimized contaminant DNA sequences, and also selected previously designed primers that successfully amplified DNA from pantherine cats [35]. Nuclear inserts of mitochondrial regions are a known problem in pantherine cats and we carefully selected primers that preferentially amplified the cytoplasmic copy [36,37]. We used either Ampli*Taq*® Gold (ABI) or KAPA2G™ Robust HotStart (KAPA Biosystems) in PCRs according to manufacturers’ guidelines, with a 90 s activation step at 95°C, followed by 45 cycles of 95°C for 45 s, T_A_ (Additional file 1: Figure S1, Additional file 2: Figure S2, Additional file 3: Table S1, Additional file 4: Table S2 and Additional file 5: Table S3) for 45 s, and 72°C for 45 s with a final extension at 72°C for 5 min. PCRs were resolved on an agarose gel. Successful amplifications were purified by the addition of exonuclease I (0.1 u/μl, Fermentas, UK), buffer (B16, Fermentas, UK) and FastAP (0.0025 u/μl, Fermentas, UK), heated at 37°C for 10 min, then inactivated at 65°C for 15 min. Purified PCRs were sequenced using BigDye® chemistry (ABI) and read on an ABI3730 at DBS Genomics (Durham, UK).

### Data analysis

Contigs were assembled in Se-Al v2.0a11 [38] and then aligned with 74 previously published lion sequences (Additional file 3: Table S1). For a subset of individuals, both *cytb* and HVR1 control region sequences were available (Additional file 3: Table S1). We did not identify any nonsense or frameshift mutations in our data. In every instance, overlapping contigs were complementary, including overlaps where diagnostic mutations were also found (Additional file 1: Figure S1, Additional file 2: Figure S2, Additional file 3: Table S1, Additional file 4: Table S2 and Additional file 5: Table S1). Both individual amplicons and assembled contigs showed greater similarity to mitochondrial sequences than to nuclear inserts of mitochondrial regions (Additional file 1: Figure S1, Additional file 2: Figure S2, Additional file 3: Table S1, Additional file 4: Table S2 and Additional file 5: Table S1).

To estimate the relationships among *cytb* haplotypes, we generated a median network [39] using the median-joining algorithm in the program Network v4.610 (http://www.fluxus-engineering.com). More published sequences were available on GenBank that consisted only of *cytb* than of *cytb* + control region. Network analysis allowed us to confirm that congruent trees were inferred using both a shorter DNA dataset containing more sequences and our longer DNA dataset containing fewer sequences.

Bayesian phylogenetic analyses were performed using BEAST v1.7 [40]. The *cytb* and control region data were concatenated (1186 bp) and the HKY substitution model was selected for each region using the Bayesian information criterion. This criterion has been shown to perform well under a variety of simulation scenarios [41]. Owing to the intraspecific nature of the data set, a strict molecular clock was assumed and a separate rate was allowed for each region. However, calibrating the molecular clock can be difficult when the data set involves deep coalescence events [42-44]. Although our data set includes ancient DNA samples, they are not old enough to provide sufficient calibrating information for the molecular clock [45]. Instead, we favour an approach that combines two sources of calibrating information. First, we employed a previous estimate of the substitution rate based on the control region of cave lions, calibrated using the radiocarbon dates of the samples [45]. This was implemented using a normal prior (mean 5.35×10^−7^ substitutions/site/year, standard deviation 2.28×10^−7^ substitutions/site/year) for the substitution rate of the control region. The mutation rate of *cytb*, relative to the control region, was estimated in the analysis. Second, we added a sequence from a cave lion, *P. leo spelaea*, which allowed us to specify a calibration for the divergence between *P. leo leo* and *P. leo spelaea*. We used a normal prior with a mean of 550,000 years and standard deviation of 25,000 years for the timing of this split, based on the appearance of the ancestral cave lion *P. leo fossilis* in the European fossil record [35,46].

An analysis using Bayes factors [47] supported a model with constant population size as the best-fitting coalescent model for the data set. We obtained posterior estimates of parameters via Markov chain Monte Carlo (MCMC) sampling. Samples were drawn every 1,000 steps over 10,000,000 MCMC steps, with the first 10% discarded as burn-in. Acceptable sampling and convergence to the stationary distribution were checked by inspection of traces using Tracer v1.5 [48].

For the phylogeographical analyses, we ran two independent MCMC simulations of 50,000,000 steps each using the discrete phylogeographical model described in Edwards *et al*. [32]. We assigned each sequence to one of seven geographical locations, broadly reflecting traditional subspecies designations [6,30]: Middle East, Asia, Central Africa, East Africa, West Africa, South Africa, and North Africa (Table 1 and Additional file 3: Table S1), and estimated rates of diffusion to and from each location. Settings for the analysis matched those described above, except we replaced the constant-size coalescent prior with the flexible Gaussian Markov random field prior (skyride plot; [49]).

### Data authenticity

We employed strict protocols to eliminate contamination and other potential artefacts [50]. We used only specimens that had been shown previously to contain authentic endogenous DNA [19,23,24,33]. DNA extractions were performed in a physically isolated laboratory separated from any modern molecular biology work, and in which no work on felids had ever been attempted. Samples were co-extracted with negative controls in extract:control ratios of between 2:1 and 9:1. We used sterile reagents, filtered pipette tips, and bleached and UV-irradiated surfaces and instruments. We set up PCRs in an isolated facility, under positive air pressure in a dedicated sterile hood, incorporating negative PCR and extraction controls. Following set-up, we manually transported PCRs to a different laboratory for thermal cycling and post-PCR work. We confirmed all putative novel mutations on the basis of at least three independent PCRs (Additional file 1: Figure S1, Additional file 2: Figure S2, Additional file 3: Table S1, Additional file 4: Table S2 and Additional file 5: Table S1). Additionally, several specimens (PL1, PL4, PL13, PL15, and PL16) produced sequences that were identical to those previously published for lions from nearby geographic regions.

## Results

**Figure 1:**
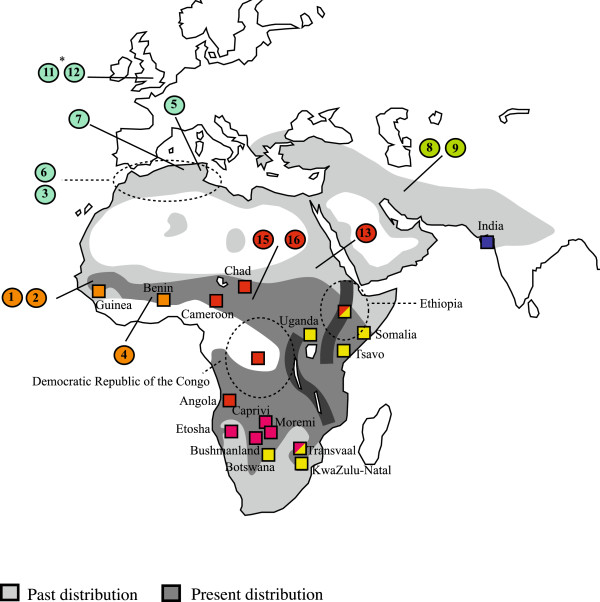
**A map of the source areas for the lion samples analysed in this study.** Numbers within circles correspond to PL numbers in Table 1. Squares correspond to sequences available on Genbank and identified in Additional file 3: Table S1. Colours of squares and circles correspond to those used in Figure 2. *PL11 and PL12 are medieval English lions that have been identified as North African lions (*P. leo leo*) based on mitochondrial HVR sequences and morphological data.

We amplified a 1051 bp fragment of *cytb* from 14 museum-preserved lions (Table 1). These lions were sampled from extinct populations in North Africa and the Middle East and endangered populations in West and Central Africa (Figure 1).

From these, we identified four new mitochondrial haplotypes: one from North Africa, one from a suspected Barbary lion present in medieval London, one from Iran, and one from Senegal. All other samples produced haplotypes identical to published data and were compatible with their geographical origin (Additional file 3: Table S1). Four of the six Barbary lions exhibited a *cytb* sequence identical to that of the extant Indian lion (Table 1 & Additional file 3: Table S1).

**Figure 2:**
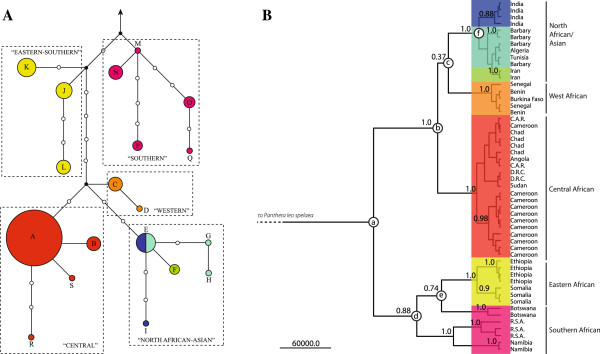
**Phylogenetic analyses of lion sequence data. A)** Median network of 1051 bp of *cytb* for all 88 lion individuals identified from GenBank plus those generated in this study. *Panthera leo spelaea* was used as an outgroup. Circles are proportional to haplotype frequencies and black circles represent hypothesized intermediate haplotypes. The number of links represent the number of mutations between haplotypes. Haplotypes are labelled from A to S and correspond to sequences labelled in Table 1 and Additional file 3: Table S1. **B)** Phylogenetic tree from a Bayesian analysis of combined *cytb* and control region data for all lion taxa where available (n = 54). Posterior probabilities of supported clades are shown at nodes. Estimates of divergence times: **(a)** 124,200 years (95% credibility: 81,800-183,500); **(b)** 61,500 years (32,700-97,300); **(c)** 51,000 years (26,600-83,100); **(d)** 81,900 years (45,700-122,200); **(e)** 57,800 years (26,800-96,600); **(f)** 21,100 years (8300–38,800). Branch colours correspond to reconstructed ancestral geographic states (Purple, South Africa; Yellow, East Africa; Orange, West Africa; Red, Central Africa; Teal, North Africa; Blue, South Asia; Green, Near-East). Tip colours correspond to origins of samples.

Network analyses of North African and Asian *cytb* sequences show a simple starburst pattern with a basal haplotype shared between North African and Indian lions (Figure 2A). Additional diversity is represented by Iranian lions that share a haplotype separated by a single synonymous mutation from the central Indian haplotype, and a wild-shot North African lion and putative Barbary lion from medieval England that differ by two and three synonymous mutations, respectively.

Bayesian phylogenetic analysis of combined *cytb* and control region data suggests a clear clustering pattern in which North African/Asian, western African, central African, eastern-southern African, and southern African lion populations are recognized, although the two Southern African clusters are not as internally coherent as the other three clades (Figure 2B). The most recent common ancestor of the five groups (node a in Figure 2B) was estimated to have existed in the Late Pleistocene (124,200 years BP, 95% HPD: 81,800-183,500). This date overlaps considerably with two published estimates for the MRCA of lions, 74,000-203,000 years BP [35] or 145,000-502,000 years BP [28]. It should be noted that the external fossil calibration prior used in our analysis has had a substantial effect on the estimates of divergence times when compared to using only the internally calibrated mutation rate from the control region data. If *Panthera leo fossilis* is a poor indicator of the split between *P.leo leo* and *P.leo spelaea*, then this would have concomitant effects on the divergence estimate [44].

The phylogeographical methods also allowed us to estimate the divergence times of individual lion clades (Figure 2B). The split between North African/Asian and West and Central African lions (node b in Figure 2B) took place approximately 61,500 years BP (95% HPD: 32,700-97,300). North African/Asian and West African lions split (node c in Figure 2B) by approximately 51,000 years BP (95% HPD: 26,600-83,100). The movement of lions out of Africa and into the Near-East/Asia appears to have occurred in two recent waves. The first expansion from North Africa (node f in Figure 2B) occurred around 21,100 years BP (95% HPD: 8,300-38,800) resulting in the major Asian lion clade, and the second migration later produced the Middle-Eastern lion clade.

**Figure 3:**
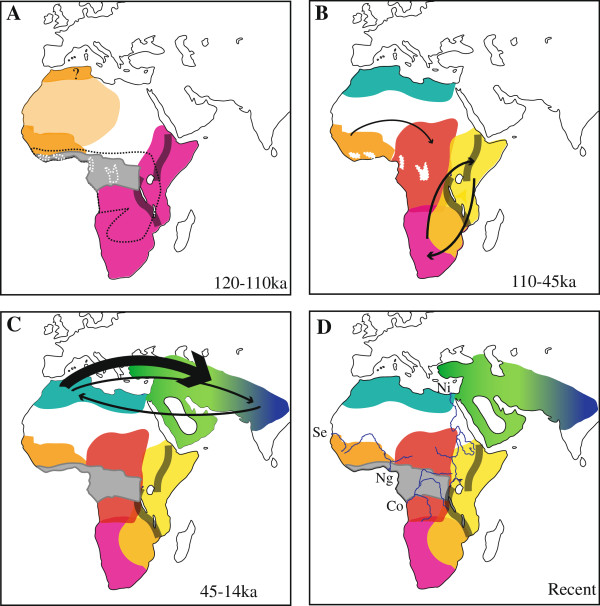
**Reconstructed distribution of the modern lion at different times.** Estimates of spatial diffusion pathways at Marine Isotope Stage (MIS) time points: **A.** MIS5 **B.** MIS4-MIS3 **C.** MIS2-MIS1 **D.** Estimated natural distribution prior to anthropogenic disturbance. Black arrows show estimated spatial diffusions, with thicknesses proportional to Bayes factors. Movement from East Africa to South Africa (4.83), from South Africa to East Africa (4.66), from West Africa to Central Africa (3.00), from North Africa to South Asia (4.37), from South Asia to North Africa (4.50), from North Africa to Middle East (21.03). Tropical rainforest is shown in light grey (present distribution), maximal extent during humid periods (black dashed line), and minimal extent during arid periods (white dashed line). The Great Rift Valley is shown in dark grey. African rivers are shown in blue. Co, Congo; Ng, Niger; Ni, Nile; Se, Senegal.

The geographical component incorporated into the Bayesian analysis also allowed us to reconstruct ancestral geographical states (Figures 2B and 3) and assign probabilities to the movements. It should be noted that these reconstructed movements are only based on maternally inherited mitochondrial DNA, and are therefore subject to future modification when biparentally inherited nuclear DNA becomes available. As suggested previously [23,28], the geographical base of the lion tree is located in sub-Saharan Africa, with movement between East and South Africa (Bayes factor: 4.83/4.66). The West African population probably acted as a source from which a population expanded into Central Africa (Bayes factor: 3.00). During the Holocene, the North African population expanded into South Asia (Bayes factor: 4.37/4.50), and then again into the Near East (Bayes factor: 21.03).

## Discussion

### Demographic and evolutionary history

We identified a Late Pleistocene origin for all five major phylogeographical groups of the modern lion (Figure 2), thus providing additional support for a single-African-origin model. This model was first proposed by Yamaguchi *et al.*[14] based on morphology, distribution, and parietal art and has been reinforced by aDNA studies of extinct Late Pleistocene lions [23,24,35]. Additional analyses of ancient DNA from historical examples of modern lions suggest that sub-Saharan Africa, with a tentatively identified focal point in eastern-southern Africa, was the likely evolutionary cradle of the modern lion [23]. The eastern-southern origin has been independently supported by Antunes *et al.*[28], who based their findings on mtDNA and lion feline immunodeficiency virus (FIV) sequences amplified from 357 modern East African, South African and Indian lions. Our results, showing longer branches amongst eastern-southern African lions (Figure 2), also support the idea that the evolutionary cradle of the modern lion was in eastern-southern Africa.

Our results suggest a very late date for the most recent common ancestor of all five phylogeographical groups. Burger *et al.*[35] previously concluded that the most recent common ancestor of the modern lion lived 74–203 thousand years BP based on mitochondrial *cytb* sequences calibrated with fossil data from *P. leo fossilis*. Antunes *et al.*[28] suggested c. 145–502 thousand years BP for a major range expansion of the modern lion, including the colonization of south-western Eurasia based on mtDNA (12S and 16S rRNA genes). Although our estimate of the divergence time overlaps with both previous estimates, we demonstrate that the modern lion exodus from Africa took place only ~21,000 years ago. This analysis has not previously been possible due to the extinction of sister populations in North Africa and Middle East, now accessible for study through ancient DNA.

Our use of statistical phylogeography and DNA sequences from purported refugial regions allows us to reconstruct the past movements of lion populations and place them in the context of palaeoclimatic evidence. Late Pleistocene Africa experienced massive, sudden fluctuations in hydrology caused by the glacial/interglacial cycle, with concomitant effects on vegetation and biome distribution [51-53]. More specifically, several episodes of rapid cycling between high humidity and aridity have been identified from the palynological record [51,53,54], with the three most recent humid phases having occurred during Marine Isotope Stage 5 (MIS5, 120-110Ka), Marine Isotope stage 3 (MIS3, 50-45Ka) and the early Holocene (10-6Ka) [55-57]. During periods of high humidity, tropical rainforest expanded across the equatorial region, and the Sahara became savanna [52,55,56]. Conversely, during periods of high aridity, tropical rainforest contracted to form isolated refugia as the Sahara expanded [51,52,54,56]. The timing and nature of these changes correlate well with inferred patterns of lion diversification and suggest a mechanism to explain the phylogeographical patterning of lions in Africa and Asia.

During the Middle Pleistocene, prior to MIS5, lions were probably widespread over Africa, occupying regions of savanna/scrub-woodland. During the humid period of MIS5, tropical rainforest expansion eastward from the Gulf of Guinea to the Great Rift Valley would have isolated southern and eastern African populations from western and northern populations, corresponding to the basal divergence among lion lineages ~124,200 years BP (95% HPD: 81,800-183,500). As aridity increased, leading into MIS4, the Sahara expanded and separated lion populations in North Africa and West Africa. This climatic phase corresponds to the bifurcation of these two populations, which is estimated to have occurred ~51,000 years (95% HPD: 26,600-83,100).

The contraction of tropical rainforests, as the continent dried, permitted the expansion of lions from West Africa into newly open biomes in central Africa, and a signal of this movement is recovered by the statistical phylogeographical analysis. At the same time, there is evidence for a complicated interaction between populations in East and South Africa, with the Rift Valley potentially acting as a partial barrier to dispersal. Lastly, our phylogenetic and demographic reconstructions provide evidence for two separate excursions into Asia by lions from North Africa, initially during the end-Pleistocene ~21,000 years BP (95% HPD: 8,300-38,800). The most recent population movement involves Iranian lions that appear to be descended from North African lions dispersing during the mid-late Holocene. The recent dates for emigration to Asia are interesting because lion remains have been found from throughout the Late Pleistocene in the Middle East [58,59]. However, the genetic data presented here only reflect the most recently arrived populations in the region. Populations that resulted from earlier expansions are unlikely to be detected since their genetic signatures would have been overwritten by those of later expansions.

The identification of well-supported phylogeographical groups of lions naturally prompts the question of what has maintained these divisions. Several obvious dispersal barriers have been proposed in previous publications, including the Great Rift Valley [27], tropical rainforests [20], and the Sahara [26], though other topographic obstacles are also likely to have played a role. The separation between West African and Central African lions occurs in the region between Benin and Cameroon, an area bisected by the Niger River (Figure 3). Similarly, the Central African lions appear to be bounded by the Nile on their eastern front, separating them from the Eastern African group (Figure 3), though the sparse sampling from this region makes identifying barriers difficult. The influence of large rivers on African mammal phylogeography has been demonstrated for chimpanzees and bonobos (*Pan* spp.), where the Niger River may act as a subspecies barrier [60]. Other African savannah ungulates, such as giraffes (*Giraffa camelopardalis*) also show a congruent pattern [5]. Similarly, large rivers appear to act as biogeographic boundaries to a certain extent, even for the jaguar (*Panthera onca*) which is renowned for its ability to swim [61]. It is probable that deserts, valleys, and rivers and watershed boundaries have all acted to maintain the phylogeographical structure of lions.

### Conservation

Our analyses recovered five major phylogeographical groups in the modern lion: North African/Asian, West African, Central African, South African, and East-South African. All of these could be designated as Evolutionarily Significant Units (ESUs) in the absence of conflicting morphological or nuclear DNA data [62] (Figure 2). This pattern is consistent with previous studies based on control region data from across the species range [23], and studies using other mitochondrial regions to examine detailed phylogeographical patterns in sub-Saharan Africa [27,28] and western-central Africa [20].

International bodies currently recognize only two lion conservation units: African and Asian lions [18] on the basis of early attempts to categorize lions using crude allozyme separation [63,64]. DNA sequence studies have questioned these widely accepted legislative conservation units because the current dichotomy does not coincide with the intraspecific phylogeny estimated using a wider sampling regime [19,20,23,28].

Our results further suggest that this dichotomy requires revision (Figure 2). The mitochondrial data clearly show that Asian lions are nested within the diversity present in Central, West, and North Africa. Perhaps *Panthera leo persica* should be treated as consubspecific with *Panthera leo leo,* or alternatively the other phylogeographical groups could be considered for elevation to the same status. Of particular concern are the central African and western African populations, which may be close to extinction, with estimates of ~800 lions in West Africa and ~900 lions in Central Africa [65]. Our data confirm the distinct nature of western African lions and the need to afford them appropriate protection [66]. At the same time, we encourage a careful approach when discussing ESUs based on mtDNA, rather than morphology or nuclear DNA, due to the scale-dependent and static nature of many units (see [23] for detailed arguments).

The close phylogenetic relationships among Barbary, Iranian, and Indian lion populations are noteworthy given their considerable geographical separation (Figures 1 and 2). Nonetheless, the extinct North African Barbary lion harboured appreciable genetic diversity prior to extirpation, including unique *cytb* haplotypes (Table 1, Figure 2A). Individuals PL3 and PL12 (Table 1) differ from the majority haplotype, though neither sample is associated with a specific provenance. PL3 was collected in “Barbary” according to museum notes, whereas PL12 was kept in the Tower of London during the 15th Century and its North African origin has only recently been identified [33]. In comparison to the Barbary lions from Tunisia and Algeria, the divergent haplotypes might reflect the historical presence of additional population subdivisions within North African lions (e.g., Atlas Mountains and Mediterranean coast populations), evidence of a large, diverse, panmictic population, or incomplete lineage sorting. Barbary lion samples with precise provenance data would be needed to resolve the issue.

The restoration of the extinct North African Barbary lion has attracted the attention of conservationists both inside and outside North Africa [19,31,67,68]. Although circumstantial evidence suggested that the Barbary lion could have survived in captivity [67,68], the most likely descendants of wild Barbary lions from the Moroccan Royal Menagerie do not appear to be (maternally) Barbary [19, this study]. However, there is a close mitochondrial relationship between the Barbary lion and the extant Indian lion, and this has been tentatively (but independently) supported by non-molecular studies [26,30].

In the tiger, another charismatic felid species, studies of ancient mitochondrial DNA have suggested a close relationship between the extinct central Asian Caspian tiger (*Panthera tigris virgata*) and the extant Amur tiger (*P. t. altaica*) [69]. This has allowed conservationists to discuss the translocation of Amur tiger stock to occupy the former range of the Caspian tiger [70], with support from the World Tiger Summit [71]. Similarly, if no examples of purebred Barbary lions can be found within the zoo population, there might be scope for restoration of the North African lion population using the closely related Indian lion.

## Conclusion

Ancient DNA, in combination with Bayesian phylogeographical analysis, has enabled us to infer a maternal evolutionary and demographic history of the lion that would be impossible using modern data alone. The inclusion of representatives of populations from North Africa, West Africa, Central Africa, and the Middle East fills major gaps in our understanding of the past and present distribution of lions and our analysis has led to two major findings. Firstly, our identification of the influence of cyclical changes in African climate, and of the expansion from refugia in western, southern and northern Africa, highlight the importance of these regions for lion diversity. Secondly, population subdivisions identified here are sufficiently divergent to warrant recognition as ESUs. Our data regarding these ESUs will greatly aid conservation planning of these important taxa over the complete natural range of modern lions.

These data can now also be used to help establish the likely provenance of zoo lions, as an assessment before potential inclusion in *ex-situ* breeding programmes. The search should now focus on nuclear markers to expand and confirm the subdivisions we have identified. The need for this will become increasingly clear as the unique lion populations of sub-Saharan Africa edge closer to endangered status.

### Availability of data

The data supporting the results of this article are available from GenBank (DNA sequences, accession nos. KJ545522-KJ545535).

## Abbreviations

MIS: Marine Isotope Stage; ESU: Evolutionarily Significant Unit; HPD: Highest Posterior Density; HVR1: Highly Variable Region 1; EDTA: Ethylenediaminetetraacetic acid; SDS: Sodium Dodecyl Sulfate; PCR: Polymerase Chain Reaction; IUCN: International Union for Conservation of Nature; MCMC: Markov Chain Monte Carlo; cytb: cytochrome b; bp: base pairs; kDa: KiloDaltons.

## Competing interests

The authors declare they have no competing interests.

## Authors’ contributions

RB, GL, and NY conceived the study. RB carried out the molecular genetic work. RB, BS, and SYWH performed data analysis. NY, RS, JC, and LW coordinated sampling of material. NY, IB, and LW interpreted the biological context of results. All authors wrote, read and approved the final manuscript.

## Supplementary Material

Additional file 1: Figure S1 Schematic of contig overlaps indicating their amplification positions along 1140bp of cytochrome *b.* Click here for file

Additional file 2: Figure S2 Neighbour-joining trees produced with PAUP* [72] of individual amplicons produced in this study, compared to known cymt (AF053040 & AF053018) and numt (AF053053 & AF053054) sequences from tiger [73]. Click here for file

Additional file 3: Table S1 List of all Genbank accessible comparative sequences and relevant information. Click here for file

Additional file 4: Table S2 List of all primers used and their annealing temperatures and lengths. Click here for file

Additional file 5: Table S3 Table of number of independent PCRs sequenced for each region. Cells with an outlined border indicate regions containing unique mutations not found in other lions. Where 2 cells share the same outlined border indicates mutation identified in region of PCR overlap and present in both contigs. Click here for file
