# Supplementary material for: Revealing the maternal demographic history of Panthera leo using ancient DNA and a spatially explicit genealogical analysis
Source: BMC Evol Biol. 2014 Apr 2;14:70. doi: 10.1186/1471-2148-14-70 (PMC3997813; doi:10.1186/1471-2148-14-70)
Supplement: Additional file 1: Figure S1 — Schematic of contig overlaps indicating their amplification positions along 1140bp of cytochrome b. [file 1471-2148-14-70-S1.pdf]

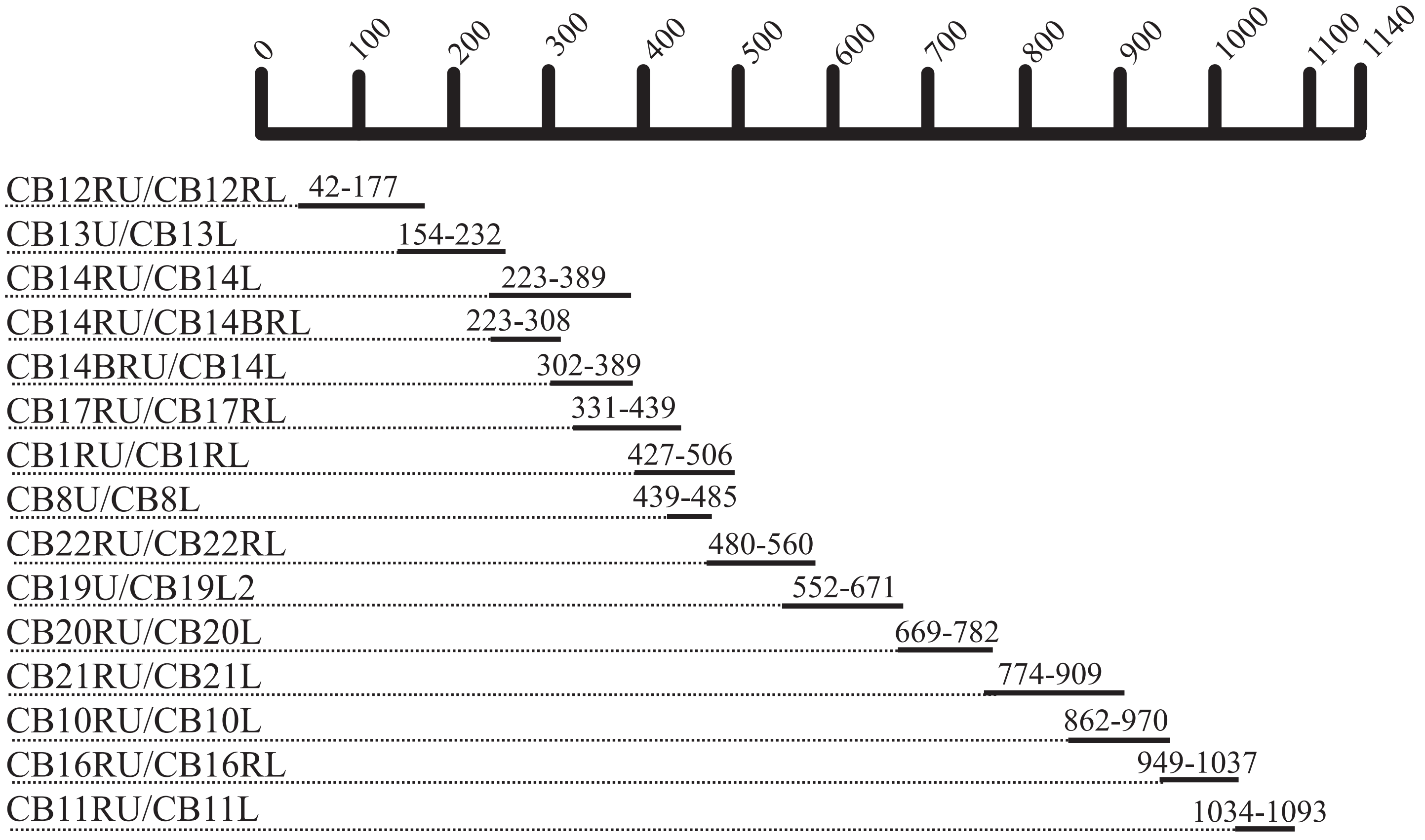
0
100
200
300
400
500
600
700
800
900
1000
1100
1140
CB12RU/CB12RL 42-177
CB13U/CB13L 154-232
CB14RU/CB14L 223-389
CB14RU/CB14BRL 223-308
CB14BRU/CB14L 302-389
CB17RU/CB17RL 331-439
CB1RU/CB1RL 427-506
CB8U/CB8L 439-485
CB22RU/CB22RL 480-560
CB19U/CB19L2 552-671
CB20RU/CB20L 669-782
CB21RU/CB21L 774-909
CB10RU/CB10L 862-970
CB16RU/CB16RL 949-1037
CB11RU/CB11L 1034-1093
