# Supplementary material for: Revealing the maternal demographic history of Panthera leo using ancient DNA and a spatially explicit genealogical analysis
Source: BMC Evol Biol. 2014 Apr 2;14:70. doi: 10.1186/1471-2148-14-70 (PMC3997813; doi:10.1186/1471-2148-14-70)

Neighbour-joining trees of individual lion amplicons compared to numt and cymt identified from tiger.

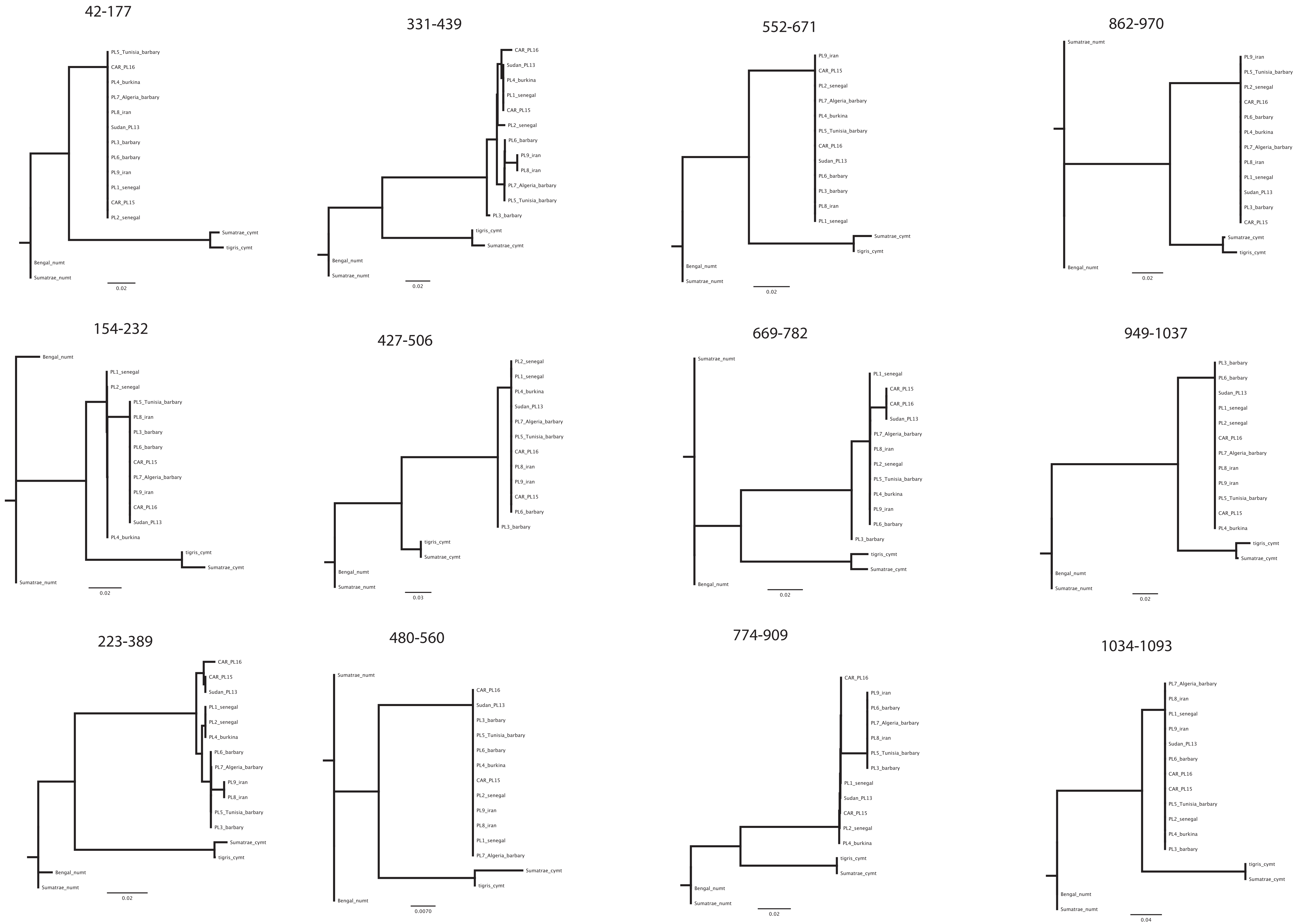

Supplement: Additional file 2: Figure S2 — Neighbour-joining trees produced with PAUP* [72] of individual amplicons produced in this study, compared to known cymt (AF053040 & AF053018) and numt (AF053053 & AF053054) sequences from tiger [73]. [file 1471-2148-14-70-S2.pdf]
