# Supplementary material for: Revealing the maternal demographic history of Panthera leo using ancient DNA and a spatially explicit genealogical analysis
Source: BMC Evol Biol. 2014 Apr 2;14:70. doi: 10.1186/1471-2148-14-70 (PMC3997813; doi:10.1186/1471-2148-14-70)
Supplement: Additional file 3: Table S1 — List of all Genbank accessible comparative sequences and relevant information. [file 1471-2148-14-70-S3.docx]

Supplementary Table 1.

| Country of Origin | ID | *Cytb* Haplotype | Genbank Accession Number | Published In | Regions | Geographic Identity in Bayesian analysis | Notes |
| --- | --- | --- | --- | --- | --- | --- | --- |
| Unknown | Unknown | A | X82300 | Arnason(1) | Cytochrome b |  |  |
| Unknown | Neuwieed Zoo | A | n/a | Hemmer(2) | Cytochrome b |  |  |
| Johannesburg Zoo | Unknown | A | AY928670 | Koepfli(3) | Cytochrome b |  |  |
| Democratic Republic of the Congo | FRITS | A | DQ018994 | Unpublished | Cytochrome b + Control Region | Central |  |
| Democratic Republic of the Congo | HELEEN | A | DQ018993 | Unpublished | Cytochrome b + Control Region | Central |  |
| Cameroon | 4 | A | AY781205 | Unpublished | Cytochrome b + Control Region | Central |  |
| Cameroon | 3 | A | AY781204 | Unpublished | Cytochrome b + Control Region | Central |  |
| Cameroon | 2 | A | AY781203 | Unpublished | Cytochrome b + Control Region | Central |  |
| Cameroon | 1 | A | AY781202 | Unpublished | Cytochrome b + Control Region | Central |  |
| Angola | PLB051 | A | AY781201 | Unpublished | Cytochrome b + Control Region | Central |  |
| Morocco (Sables-d’Olonne Zoo) | P96030 | A | AY781196 | Unpublished | Cytochrome b + Control Region |  | Control Region is highly similar to NuMt |
| Morocco (Sables-d’Olonne Zoo) | P88017 | A | AY781195 | Unpublished | Cytochrome b + Control Region |  | Control Region is highly similar to NuMt |
| Chad | 4 | A | AY781200 | Unpublished | Cytochrome b + Control Region | Central |  |
| Morocco (Rabat Zoo) | 077_298_006 | A | DQ022301 | Unpublished | Cytochrome b |  |  |
| Morocco (Rabat Zoo) | 077_365_350 | A | DQ022300 | Unpublished | Cytochrome b |  |  |
| Morocco (Rabat Zoo) | 077_546_597 | A | DQ022299 | Unpublished | Cytochrome b |  |  |
| Morocco (Rabat Zoo) | 077_616_888 | A | DQ022298 | Unpublished | Cytochrome b |  |  |
| Morocco (Rabat Zoo) | 077_633_073 | A | DQ022297 | Unpublished | Cytochrome b |  |  |
| Morocco (Rabat Zoo) | 077_360_082 | A | DQ022296 | Unpublished | Cytochrome b |  |  |
| Morocco (Rabat Zoo) | 077_769_520 | A | DQ022295 | Unpublished | Cytochrome b |  |  |
| Morocco (Rabat Zoo) | 077_551_297 | A | DQ022294 | Unpublished | Cytochrome b |  |  |
| Morocco (Rabat Zoo) | 077_616_301 | A | DQ022293 | Unpublished | Cytochrome b |  |  |
| Morocco (Rabat Zoo) | 077_521_637 | A | DQ022292 | Unpublished | Cytochrome b |  |  |
| Morocco (Rabat Zoo) | 077-345-639 | A | DQ022291 | Unpublished | Cytochrome b |  |  |
| Cameroon (Waza) | 4e | A | GU131175 | Bertola(4) | Cytochrome b + Control Region | Central | Waza National Park |
| Cameroon (Waza) | 4d | A | GU131174 | Bertola(4) | Cytochrome b + Control Region | Central | Waza National Park |
| Cameroon (Waza) | 4c | A | GU131173 | Bertola(4) | Cytochrome b + Control Region | Central | Waza National Park |
| Cameroon (Waza) | 4b | A | GU131172 | Bertola(4) | Cytochrome b + Control Region | Central | Waza National Park |
| Cameroon (Waza) | 4a | A | GU131171 | Bertola(4) | Cytochrome b + Control Region | Central | Waza National Park |
| Cameroon (Benoue) | 3b | A | GU131170 | Bertola(4) | Cytochrome b + Control Region | Central | Benoue National Park |
| Chad | 3 | B | AY781199 | Unpublished | Cytochrome b + Control Region | Central | Zakouma National Park |
| Chad | 2 | B | AY781198 | Unpublished | Cytochrome b + Control Region | Central | Zakouma National Park |
| Chad | 1 | B | AY781197 | Unpublished | Cytochrome b + Control Region | Central | Zakouma National Park |
| Cameroon (Benoue) | 3a | B | GU131169 | Bertola(4) | Cytochrome b + Control Region | Central | Benoue National Park |
| ? | TBZ2 | C | DQ022303 | Unpublished | Cytochrome b |  |  |
| ? | TBZ1 | C | DQ022302 | Unpublished | Cytochrome b |  |  |
| Benin | 1b | C | GU131165 | Bertola(4) | Cytochrome b + Control Region | West |  |
| Benin | 1a | C | GU131164 | Bertola(4) | Cytochrome b + Control Region | West |  |
| Guinea | 1 | C | DQ018996 | Unpublished | Cytochrome b |  |  |
| India | 5031 Planckendael Zoo | E | AY781206 | Unpublished | Cytochrome b + Control Region | Asia |  |
| Gir Zoo | 5c Diergaarde Blijdorp Zoo | E | GU131178 | Bertola(4) | Cytochrome b + Control Region | Asia |  |
| Gir Zoo | 5b Diergaarde Blijdorp Zoo | E | GU131177 | Bertola(4) | Cytochrome b + Control Region | Asia |  |
| Gir Zoo | 5a Diergaarde Blijdorp Zoo | E | GU131176 | Bertola(4) | Cytochrome b + Control Region | Asia |  |
| India | PLP1 | E |  | Burger(5) | Cytochrome b |  |  |
| India | PLP2 | E |  | Burger(5) | Cytochrome b |  |  |
| Indian? | Brookfield Zoo | I | AF053052 | Cracraft(6) | Cytochrome b |  |  |
| Uganda | UGD80 | J | AF384809 | Dubach(7) | Cytochrome b |  |  |
| Somalia | Amiri | J | DQ018995 | Unpublished | Cytochrome b + Control Region | East |  |
| Unknown | S1 | J | DQ022290 | Unpublished | Cytochrome b |  |  |
| Somalia Zoo | 7b | J | GU131182 | Bertola(4) | Cytochrome b + Control Region | East |  |
| Somalia Zoo | 7a | J | GU131181 | Bertola(4) | Cytochrome b + Control Region | East |  |
| RSA (KwaZulu-Natal) | UMF102 | K | AF384818 | Dubach(7) | Cytochrome b |  | Hluhluwe-Umfolozi Park |
| RSA (Transvaal) | KPR98 | K | AF384816 | Dubach(7) | Cytochrome b |  | Kapama Game Reserve |
| Kenya (Tsavo) | TSV355 | K | AF384817 | Dubach(7) | Cytochrome b |  | Tsavo East National Park |
| Botswana Zoo | 2c | K | GU131168 | Bertola(4) | Cytochrome b + Control Region | South |  |
| Botswana Zoo | 2b | K | GU131167 | Bertola(4) | Cytochrome b + Control Region |  | Only partial control region |
| Botswana Zoo | 2a | K | GU131166 | Bertola(4) | Cytochrome b + Control Region | South |  |
| Ethiopia | Laith | L | AY781210 | Unpublished | Cytochrome b + Control Region | East |  |
| Ethiopia | 0001C8278B | L | AY781209 | Unpublished | Cytochrome b + Control Region | East |  |
| Ethiopia | 0001C7F021 | L | AY781208 | Unpublished | Cytochrome b + Control Region | East |  |
| Ethiopia | 0001C70EA1 | L | AY781207 | Unpublished | Cytochrome b + Control Region | East |  |
| Namibia (Etosha) | ETO65 | M | AF384811 | Dubach(7) | Cytochrome b |  | Etosha National Park |
| Botswana (Moremi) | BOT16 | N | AF384815 | Dubach(7) | Cytochrome b |  | Moremi Game Reserve |
| Namibia (Etosha) | ETO76 | N | AF384812 | Dubach(7) | Cytochrome b |  | Etosha National Park |
| Namibia (Bushmanland) | BML22 | N | AF384813 | Dubach(7) | Cytochrome b |  |  |
| Namibia (Caprivi Strip) | CAP30 | N | AF384814 | Dubach(7) | Cytochrome b |  |  |
| RSA (Transvaal) | PSS993 | O | AF384810 | Dubach(7) | Cytochrome b |  | Sabi Sands Region |
| Kruger Zoo (Transvaal) | 8b | O | GU131184 | Bertola(4) | Cytochrome b + Control Region | South | Timbavati Games Reserve |
| Kruger Zoo (Transvaal) | 8a | O | GU131183 | Bertola(4) | Cytochrome b + Control Region | South | Timbavati Games Reserve |
| Namibia Zoo | 6b | P | GU131180 | Bertola(4) | Cytochrome b + Control Region | South |  |
| Namibia Zoo | 6a | P | GU131179 | Bertola(4) | Cytochrome b + Control Region | South |  |
| Kruger Zoo | 8c | Q | GU131185 | Bertola(4) | Cytochrome b + Control Region | South | Timbavati Games Reserve |
| San Diego Zoo (KB13712) | PLE krugeri | R | HM107681 | Davis(8) | Cytochrome b |  |  |
| Ethiopia | Addis Ababa Zoo | S | JX023542 | Bruche(9) | Cytochrome b |  | Unknown ancestry |

References:

1. Arnason U, Bodin K, Gullberg A, Ledje C, Mouchaty S. A molecular view of pinniped relationships with particular emphasis on the true seals. Journal of Molecular Evolution 1995;40:78-85.

2. Hemmer H, Burger J. Genetic Confirmation of the Unique Nature of the Zoo Population of the Barbary Lion. International Zoo News 2005;52(4):218-223.

3. Koepfli K-P, Jenks SM, Eizirik E, Zahirpour T, Van Valkenburgh B, Wayne RK. Molecular systematics of the Hyaenidae: Relationships of a relictual lineage resolved by a molecular supermatrix. Molecular Phylogenetics and Evolution 2006;38:603-620.

4. Bertola LD, van Hooft WF, Vrieling K, Uit de Weerd DR, York DS, Bauer H, et al. Genetic diversity, evolutionary history and implications for conservation of the lion (Panthera leo) in West and Central Africa. Journal of Biogeography 2011;38(7):1356-1367.

5. Burger J, Rosendahl W, Loreille O, Hemmer H, Eriksson T, Gotherstrom A, et al. Molecular phylogeny of the extinct cave lion *Panthera leo spelaea*. Molecular Phylogenetics and Evolution 2004;30(3):841-849.

6. Cracraft J, Feinstein J, Vaughn J, Helm-Bychowski K. Sorting out tiger (*Panthera tigris*): mitochondrial sequences, nuclear inserts, systematics, and conservation genetics. Animal Conservation 1998;1:139-150.

7. Dubach J, Patterson BD, Briggs MB, Venzke K, Flamand J, Stander P, et al. Molecular genetic variation across the southern and eastern ranges of the African lion, *Panthera leo*. Conservation Genetics 2005;6:15-24.

8. Davis BW, Li G, Murphy WJ. Supermatrix and species tree methods resolve phylogenetic relationships amoung the big cats, Panthera (Carnivora: Felidae). Molecular Phylogenetics and Evolution 2010.

9. Bruche S, Gusset M, Lippold S, Barnett R, Eulenberger K, Junhold J, et al. A genetically distinct lion (Panthera leo) population from Ethiopia. European Journal of Wildlife Research 2012.
