# Supplementary material for: Revealing the maternal demographic history of Panthera leo using ancient DNA and a spatially explicit genealogical analysis
Source: BMC Evol Biol. 2014 Apr 2;14:70. doi: 10.1186/1471-2148-14-70 (PMC3997813; doi:10.1186/1471-2148-14-70)
Supplement: Additional file 4: Table S2 — List of all primers used and their annealing temperatures and lengths. [file 1471-2148-14-70-S4.docx]

Supplementary Table 2.

| Primer Name | Sequence 5’-3’ | Extension T_A_ | Length(bp) | Partner | Sequence 5’-3’ |
| --- | --- | --- | --- | --- | --- |
| CB12RU | CACACCCCCTTGTCAAAATTATT | 50 | 180 | CB12RL | TGACTGATGAGAAAGCGGTTAT |
| CB13U* | AAATTCTCACCGGCCTCTTTCTA | 52 | 124 | CB13L* | TTGGCGTGTAGGTACCGGATAA |
| CB14RU | CACATTTGCCGCGATGTAAAY | 50 | 209 | CB14L* | TGGCCCCACGGTAAGACATAT |
| CB14RU | CACATTTGCCGCGATGTAAAY | 56 | 132 | CB14BRL | TTTCTGAGAAAGTATAGGAGCCATAG |
| CB14BRU | CCTATACATGCATGTAGGACGAG | 56 | 131 | CB14L* | TGGCCCCACGGTAAGACATAT |
| CB17RU | TACTATGGCTCCTATACYTTCTCA | 50 | 156 | CB17RL | TGGGATTGCTGATAGGAGATTRG |
| CB1RU | CCAAATATCCTTTTGAGGTGC | 56 | 118 | CB1RL | TCAGGGTGGCTTTGTCTAC |
| CB8U* | TTTTGAGGTGCAACTGTAATC | 46 | 87 | CB8RL | GAAGCCTCCTCAGATYCAC |
| CB22RU | CATACATCGGRGCCGACCT | 52 | 124 | CB22RL | GATTCTTTGCCTTCCACTTCAT |
| CB19U* | GATTCTTTGCCTTCCACTTCAT | 50 | 167 | CB19L2* | AAGGCCTAGGATATCTTTGATTGTA |
| CB20RU | CAGATAAAATTCCATTCCATCCATA | 50 | 157 | CB20L* | TGGGGAGGGGTGCTTAGA |
| CB21RU | ATCCCGAYAACTATACCCC | 46 | 167 | CB21L* | GAGGGCAGGGATAATTGCTAAG |
| CB10RU | CTCCGATCTATTCCCAAYAAACT | 46 | 151 | CB10L* | CCGCTACTAGGAATCAGAATA |
| CB16RU | CCAAACAGCGAGGAWTAATG | 46 | 128 | CB16RL | TTGGCCRATGGTGATGAAG |
| CB11RU | GTGGCCARCCTGTAGAAC | 56 | 97 | CB11L* | ATGCCTGAGATGGGTATTA |

*From Burger et al.
